# Establishing an effective antimicrobial stewardship program at four secondary-care hospitals in India using a hub-and-spoke model

**DOI:** 10.1017/ash.2023.171

**Published:** 2023-06-09

**Authors:** Naveena Gracelin Princy Zacchaeus, Prasannakumar Palanikumar, Hanna Alexander, Jemin Webster, Indu K. Nair, Mahima Sadanshiv, Rincy Merlin Thomas, Divya Deodhar, Prasanna Samuel, Priscilla Rupali

**Affiliations:** 1 Department of Infectious Diseases, Christian Medical College, Vellore, Tamilnadu, India; 2 Baptist Christian Hospital, Tezpur, Assam, India; 3 Bangalore Baptist Hospital, Bangalore, Karnataka, India; 4 Padhar Hospital, Padhar, Madhya Pradesh, India; 5 Christian Fellowship Hospital, Oddanchatram, Tamilnadu, India; 6 Department of Biostatistics, Christian Medical College, Vellore, Tamilnadu, India

## Abstract

**Background:**

The high burden of antimicrobial resistance in India necessitates the urgent implementation of antimicrobial stewardship programs (ASPs) in all healthcare settings in India. Most ASPs are based at tertiary-care centers, with sparse data available regarding the effectiveness of an ASP in a low-resource primary/secondary-care setting.

**Methods:**

We adopted a hub-and-spoke model to implement ASPs in 4 low-resource, secondary-care healthcare settings. The study included 3 phases measuring antimicrobial consumption data. In the baseline phase, we measured days on antimicrobial therapy (DOTs) with no feedback provided. This was followed by the implementation of a customized intervention package. In the postintervention phase, prospective review and feedback were offered by a trained physician or ASP pharmacist, and days of therapy (DOT) were measured.

**Results:**

In the baseline phase, 1,459 patients from all 4 sites were enrolled; 1,233 patients were enrolled in the postintervention phase. Both groups had comparable baseline characteristics. The key outcome, DOT per 1,000 patient days, was 1,952.63 in the baseline phase and significantly lower in the post-intervention period, at 1,483.06 (*P* = .001). Usage of quinolone, macrolide, cephalosporin, clindamycin, and nitroimidazole significantly decreased in the postintervention phase. Also, the rate of antibiotic de-escalation was significantly higher in the postintervention phase than the baseline phase (44% vs 12.5%; *P* < .0001), which suggests a definite trend toward judicious use of antibiotics. In the postintervention phase, 79.9% of antibiotic use was justified. Overall, the recommendations given by the ASP team were fully followed in 946 cases (77.7%), partially followed in 59 cases (4.8%), and not followed in 137 cases (35.7%). No adverse events were noted.

**Conclusion:**

Our hub-and-spoke model of ASP was successful in implementing ASPs in secondary-care hospitals in India, which are urgently needed.

Antimicrobial resistance (AMR) poses a grave danger to global health.^1^ The World Health Organization (WHO) has declared that AMR is 1 of the 10 greatest global public health threats facing humanity, with 10 million predicted deaths by the year 2050.^2^ Antibiotic overuse and abuse are significant drivers for the development of AMR. India was the highest consumer of antibiotics in 2010, with 12×10^9^ units (10×10^7^ units per person).^3,4^ Cheap and easy access to antibiotics over the counter with inadequate health infrastructure is responsible for this increased use.^5^ Hence, India faces a significant problem with rising antimicrobial resistance rates.^6^ In the last decade in India, multidrug-resistant infections have significantly increased; resistance has increased to third-generation cephalosporin (ESBL) and carbapenems (CRE) in *Escherichia coli* and *Klebsiella pneumoniae* isolates, carbapenem-resistant *Acinetobacter baumannii* (CRAB). Also, fluoroquinolone or nalidixic acid resistance (NARST) has increased in *Salmonella typhi* as has methicillin-resistant *Staphylococcus aureus* (MRSA).^6,7^

Because human antimicrobial use and abuse is a significant driver for antimicrobial resistance, optimizing antimicrobial therapy via contextual and relevant antimicrobial stewardship (ASP) programs are urgently needed.^5^ Determinants and drivers of AMR may be different among primary, secondary, and tertiary-care settings,^8^ and it is likely that different approaches may need to be employed to curb inappropriate use. Successful ASP interventions are well studied in tertiary-care settings; however, the majority of the Indian population has access only to primary and secondary-care settings, where appropriate and relevant ASP interventions are lacking.^9^

Primary and secondary-care settings often have poor diagnostic services and are less likely to have well-established stewardship programs, but ironically, they have easy access to broad-spectrum, newer antibiotics.

The challenges in implementing antimicrobial stewardship at resource-limited secondary-care hospitals are the nonavailability of antibiogram, lack of microbiological support, busy physicians with no access or time to update knowledge, dearth of ASP champions to lead change compounded by administrative noncooperation or apathy, and lack of trained infectious disease specialists or pharmacists.^10^

In this study, we evaluated the effectiveness of the “hub and spoke model” of ASPs in 4 secondary-care hospitals (facilitated by a tertiary-care center) to bridge these specific gaps: training physicians in antimicrobial stewardship, strengthening diagnostic services, creating an antibiogram, and developing relevant facility-specific antimicrobial guidelines.

## Methods

We selected 4 secondary-care hospitals from different geographic regions in India: Padhar Hospital in Madhya Pradesh, Baptist Christian Hospital in Assam, Christian Fellowship Hospital in Tamilnadu, and Bangalore Baptist Hospital in Karnataka. The facilitating host center was Christian Medical College, Vellore, Tamil Nadu. The study had 3 phases: baseline assessment, intervention, and postintervention.

A gap and needs analysis of these centers before implementation of the intervention showed 4 main lacunae: (1) lack of training for healthcare professionals on judicious antibiotic prescribing; (2) inadequate laboratory diagnostic facilities and interpretation; (3) absence of antibiogram; and (4) nonavailability of standard treatment guidelines for infections. Administrative buy-in was sought at the inception of the project. Local administrators were supportive of this quality improvement project to benefit not only the patients but also hospitals seeking accreditation in the long run.

### Baseline phase

In the baseline phase, patterns of antimicrobial use, common indications for using antibiotics, and prevalence of multidrug-resistant organisms in the 4 chosen centers were recorded along with the antimicrobial consumption data in days of therapy per 1,000 patient days. The patients on selected antibiotics for at least 48 hours in general wards were recruited consecutively for 6 months. The chosen antimicrobials of interest in this study were polymyxins, carbapenems, β-lactam/β-lactamase inhibitor combinations, fluoroquinolones, macrolides, third- and fourth-generation cephalosporins, sulphonamides, tetracyclines, glycopeptides, aminoglycosides, penicillins, oxazolidinones, nitroimidazole, nitrofurantoin, clindamycin, and others.

### Intervention phase

This phase included the implementation of an intervention package that was customized to the needs of each secondary-care hospital. Components included training of study physicians in general infectious diseases through a blended distance learning program over a year. This meticulously designed course included online modules as well as face-to-face contact sessions with certification after completion of the same. The training program included 12 modules with a syndromic approach to infections of various organ systems with an emphasis on early diagnosis, appropriate antimicrobial treatment, diagnostic stewardship, and infection prevention and control. Completion of this training course required establishing an ASP in the hospital.

The study physicians were trained along with 1 additional member from each center (either a pharmacist or a nurse) for formulation and implementation of the ASP throughout the study period along with support from existing personnel. The physician was primarily responsible for providing the recommendations during the intervention period and for ensuring that all components for an effective ASP were implemented.

The programs were also assisted with the augmentation of the existing laboratory skills by training personnel at a central facility (CMC Vellore) and the development of an antibiogram based on their local hospital microbial resistance patterns via WHONET. Although observations regarding inadequate infrastructure were made during the study period, we provided only technical input to streamline and optimizing the existing infrastructure, and we encouraged local administration to support any required upgrades to ensure sustainability (Table 7, ASP intervention package). In addition, 100 consecutive infectious disease diagnoses in which antibiotics were prescribed were recorded for the creation of local clinical practice algorithms for each center by the study physician. Thus, we created locally relevant antibiotic guidelines and policies based on the local infectious disease spectrum and antibiogram.

### Postintervention phase

The effectiveness of this ASP intervention package was measured in the follow-up phase over 8 months. The study physicians in their respective centers assessed all eligible inpatients (those on antibiotics for >48 hours) and adopted prospective audit and feedback to optimize antimicrobial therapy. Syndromic diagnosis at admission, empirical antibiotic initiation, microbiologic confirmation of diagnosis, appropriateness of antibiotic usage, and compliance with recommendations were assessed. Antibiotic usage by DOTs per 1,000 patient days and indicators were compared between the baseline and postintervention phases.

### Statistical analysis

For continuous data, such as age and length of stay, the descriptive statistics n, mean, and SD was calculated, and for nonnormally distributed data, median and IQR. All categorical variables have been represented as numbers and percentages. Days of therapy for study antimicrobials per 1,000 patient days were calculated for the baseline and postintervention phases and were compared using a test for proportions. Based on the normality of data, the parametric *t* test or nonparametric Mann-Whitney *U* test was applied to find the difference between groups. The χ^2^ or Fisher exact test was applied to find the association between categorical variables. All tests were 2-sided at an α = .05 level of significance. All analyses were conducted using STATA version 16.0 software (StataCorp, College Station, TX).

## Results

### Study participants

In total, 1,459 patients and 1,233 patients were enrolled in the baseline and postintervention phases, respectively. The mean age and sex distribution were similar in both phases. Patients with chronic respiratory disorders predominated in the baseline phase compared to the postintervention phase (12.9 % vs 6.3 %; *P* = .001). Most patients were admitted to medical units, followed by surgery, orthopedics, obstetrics, and gynecology. The most common source of infection was the lower respiratory tract (30% vs 19.4 %; *P* = .001), followed by genitourinary (15.4 % vs 21.2 %; *P* = .001), and undifferentiated fever (9.3 % vs 4.2 %; *P* = .001). Community-acquired infections (74.8 vs 68.8 %; *P* = .001) were more frequent than healthcare-associated infections (3.4% vs 1.4%; *P* = .001) or hospital-acquired infections (1.7 vs 2.2; *P* = .390) (Table 1).

**Table 1.** Baseline Characteristics of Patients Enrolled a During the Baseline Phase and During the Postintervention Phase

Variables	Overall, No. (%)	Baseline Phase, No. (%)	Postintervention Phase, No. (%)	*P* Value
Total no. of patients	2,692	1,459 (54.2)	1,233 (45.8)	
Age, mean y (SD)	49.1 (18.4)	49.3 (18.9)	48.9 (17.8)	.692
Sex, male	1,341 (50.5)	730 (50.7)	611 (50.1)	.868
**Comorbidity**				
Diabetes	736 (27.3)	382 (26.2)	354 (28.7)	.143
Hypertension	626 (23.2)	316 (21.7)	310 (25.1)	**.033**
Tuberculosis	55 (2.1)	32 (2.2)	23 (1.9)	.541
Malignancy	110 (4.1)	38 (2.6)	72 (5.8)	**<.001**
Postsurgical	94 (3.5)	69 (4.7)	25 (2.0)	**<.001**
Coronary artery disease	145 (5.4)	58 (4.0)	87 (7.1)	**<.001**
Chronic respiratory disease	264 (9.8)	187 (12.9)	77 (6.3)	**<.001**
Chronic kidney disease	160 (5.9)	91 (6.3)	69 (5.6)	.475
Chronic liver disease	107 (3.9)	58 (4.0)	49 (4.0)	.997
Prior steroid use	27 (1.0)	3 (0.2)	24 (2.0)	**<.001**
Other diseases	366 (13.6)	197 (13.5)	169 (13.7)	.871
**Primary source of infection**				
None, undetermined	547 (20.3)	238 (16.3)	309 (25.1)	**<.001**
Central nervous system	67 (2.5)	36 (2.5)	31 (2.5)	.952
Skin wound	356 (13.3)	200 (13.8)	156 (12.7)	.410
Endocarditis	6 (0.2)	2 (0.1)	4 (0.3)	.305
Undifferentiated fever	187 (6.9)	135 (9.3)	52 (4.2)	**<.001**
Upper respiratory	49 (1.8)	32 (2.2)	17 (1.4)	.113
Genitourinary	485 (18.1)	224 (15.4)	261 (21.2)	**<.001**
Intra-abdominal	433 (16.1)	221 (15.2)	212 (17.2)	.158
Lower respiratory	676 (25.2)	437 (30.0)	239 (19.4)	**<.001**
Bone related	35 (1.3)	18 (1.2)	17 (1.4)	.750
Others	109 (4.1)	81 (5.6)	28 (2.3)	**<.001**
**Origin of onset of infection**				
Community onset	1,936 (72.1)	1,088 (74.8)	848 (68.8)	**.001**
Healthcare associated	66 (2.5)	49 (3.4)	17 (1.4)	**.001**
Hospital acquired	52 (1.9)	25 (1.7)	27 (2.2)	.390

### Antibiotic usage

All antibiotics prescribed across all 4 study sites during the study period were evaluated and included in the analysis. The most widely used antibiotics in the baseline period were cephalosporins (21%), β-lactam/β-lactamase inhibitors (21%), and nitroimidazoles (15%), followed by macrolides (10%), carbapenems (8%), and quinolones (6%). During the postintervention period, β-lactam/β-lactamase inhibitors (30%) were the most widely used antibiotics, followed by cephalosporin (16%), nitroimidazole (13%), carbapenems (10%), and macrolides (8%). The key outcome, DOT per 1,000 patient days, was 1,952.63 in the baseline phase and significantly decreased in the postintervention period, at 1,483.06 (*P* = .001). Antibiotic use for the following antibiotics remarkably decreased in the postintervention group compared to baseline: quinolones (536 vs 1031; *P* ≤ .001), macrolides (984 vs 1,555; *P ≤* .001), cephalosporin (2,060 vs 3,348; *P* ≤ .001), clindamycin (60 vs 94; *P* = .0022), and nitroimidazole (1,610 vs 2,323; *P* ≤ .001) (Table 3). The use of β lactam/β-lactamase inhibitor combinations increased compared with the baseline period (439.62 vs 408.02 DOT per 1,000 patient days; *P* ≤ .001), but carbapenem and colistin usage remained stable.

Based on an overall assessment by the study team, antibiotic use was justified among 64.5% in the baseline phase and 79.9% in the postintervention phase. A comparison of indications for antibiotic initiation showed the following proportions between the baseline versus postintervention phases, respectively: definite infection was seen (35.3% vs 45.3%; *P* = .001); probable infection (41.7% vs 33.3%; *P* = .001); and prophylaxis (23.2% vs 21.2%; *P* =.234). Notably, antibiotic use increased for a definite infection and declined for a probable infection and prophylaxis, suggesting a trend for judicious use of antibiotics for a microbiologically confirmed infection.

During the postintervention phase, the intervention rate was 38%, deﬁned as the number of courses of therapy in which a modification was recommended divided by the total number of courses. Overall, the recommendations given by the ID team were fully accepted in 946 cases (77.7%), partially followed in 59 cases (4.8%), and not followed in 137 cases (35.7%). De-escalation, recommended by the ID team in 50 patients, was accepted in 22 cases (44%). Similarly, discontinuation, recommended in 315 patients, was accepted in 204 cases (65.8%).

Stoppage of redundant cover was recommended in 37 patients and was done in 9 cases (24.3%). Modification according to susceptibility was recommended in 116 patients and was done in 54 cases (46.6%). Continuing the prescribed antimicrobial therapy was recommended in 763 cases, and the recommendation was followed in 704 cases (93.9%) (Tables 5 and 6). The rate of de-escalation was significantly higher in the postintervention phase than in the baseline phase (44% vs 12.5%; *P* < .0001), which suggests a definite trend toward the judicious use of antibiotics (Table 3). Common reasons for unjustified antibiotic use in the postintervention phase included wrong choice (59.75%), duration (39.4%), route of administration (33.3%), and no clinical indication (13.9%) (Table 2).

**Table 2.** Overview of Antibiotic Therapy Usage

Variables	Overall	Baseline Phase, No. (%)	Postintervention Phase, No. (%)	*P* Value
**Indication for antimicrobial therapy**				
Definite infection	1,074 (39.9)	515 (35.3)	559 (45.3)	**<.001**
Probable infection	1,018 (37.8)	608 (41.7)	410 (33.3)	**<.001**
Prophylaxis	600 (22.3)	338 (23.2)	262 (21.2)	.234
**Antibiotic therapy status after 48 hours of study enrolment or discharge in baseline phase^a^**				
Inappropriate prophylaxis		2 (0.1)		
Continued antibiotic therapy		787 (53.9)		
De-escalated antibiotic therapy		183 (12.5)		
Discontinued antibiotic therapy		63 (4.3)		
Escalated antibiotic therapy		114 (7.8)		
Changed IV to oral therapy		443 (30.4)		
Stopped redundant cover		0 (0)		
Discharge with antibiotics		324 (22.2)		
**Justification for antibiotic use**		Baseline Phase, No. (%)	Postintervention Phase, No. (%)	*P* Value
Justified Unjustified		940 (64.5) 518 (35.5)	980 (79.9) 246 (20.1)	**<.001**
**Reason for unjustified use of antibiotics in the postintervention phase**				
Inappropriate choice		147 (11.9)		
Inappropriate duration		97 (7.9)		
Inappropriate mode of administration		82 (6.7)		
**Reason for inappropriate choice of antibiotics**				
Narrow-spectrum antibiotics available		47 (3.8)		
Clinically not indicated		104 (8.4)		
Redundant cover		8 (0.6)		
Other		5 (0.4)		

a Numbers will not add up to the total number of patients because a single patient may have had multiple options.

**Table 3.** Days of Therapy (DOT) of Antimicrobials at Baseline and During the Postintervention Phase

Antibiotics	Baseline		Postintervention		*P* Value
	DOT	DOT/1,000 Patient Days	DOT	DOT/1,000 Patient Days	
**Access group**					
Sulfonamides	140	17.18	58	6.77	**<.001**
Tetracyclines	604	74.12	382	44.62	**<.001**
Aminoglycosides	680	83.44	670	78.25	.2349
Nitroimidazole	2,323	285.06	1,610	188.04	**<.001**
Nitrofurantoin	32	3.93	57	6.66	**.0154**
Clindamycin	94	11.53	60	7.01	**.0022**
Penicillins	475	58.29	294	34.34	**<.001**
**Watch group**					
B-lactams/β-LIs	3,325	408.02	3,764	439.62	**<.001**
Quinolones	1,031	126.52	536	62.60	**<.001**
Macrolides	1,555	190.82	984	114.93	**<.001**
Cephalosporins	3,348	410.85	2,060	240.59	**<.001**
Glycopeptides	245	30.06	164	19.15	**<.001**
**Reserve group**					
Colistin/Polymyxin	506	62.09	475	55.48	.0690
Carbapenems	1,232	151.18	1,282	149.73	.7929
Oxazolidinone	317	38.90	283	33.05	**.0423**
Others	5	0.61	19	2.22	**.0062**
Total	15,912	1,952.63	12,698	1,483.06	**<.001**

### MDRO infection

The overall prevalence of multidrug-resistant organisms (MDROs) was 14.5% in the baseline phase and 11.3% in the postintervention phase. The prevalences of ESBL in the postintervention and baseline phases, respectively, were 10.6% versus 8.6%. The prevalences of MRSA in the postintervention and baseline phases, respectively, were 3% versus 0.7%. The prevalences of CRO in the postintervention and baseline phases, respectively, were 1.1% versus 2%. The prevalences of VRE in the postintervention and baseline phases, respectively, were 0% versus 0.1% (*P ≤* .001) (Table 4).

**Table 4.** Secondary Outcomes

Variable	Baseline Phase (n=1,459), No. (%)	Postintervention Phase (n=1,233), No. (%)	*P* Value
De-escalation rate	186 (12.5)	22 (44.0)	**<.001**
**Mortality**	59 (4)	32 (2.5)	.193
Infectious	36 (61.0)	14 (43.758)	
Noninfectious	8 (13.6)	4 (12.5)	
Both	15 (25.4)	14 (43.75)	
Length of stay, median d (IQR)	5.0 (3.0–8.0)	6.0 (4.0–9.0)	**<.001**
**Prevalence of MDROs**			**.001**
Extended-spectrum β lactamase (ESBL)	89 (10.6)	65 (8.6)	
Vancomycin-resistant *Enterococcus* (VRE)	0 (0.0)	1 (0.1)	
Carbapenem-resistant Enterobacteriaceae (CRE)	9 (1.1)	15 (2.0)	
Methicillin-resistant *Staphylococcus aureus* (MRSA)	25 (3.0)	5 (0.7)	
None	720 (85.4)	674 (88.7)	
**Unintended consequences**			
No reaction	1,455 (99.7)	1,221 (99.0)	**.023**
Diarrhea	0 (0.0)	7 (0.6)	**.004**

Note. IQR, interquartile range; MDRO, multidrug-resistant organism.

**Table 5.** Acceptance of Recommendation Given by the Infectious Disease Physician During the Postintervention Phase

Type of Recommendation	Recommendation Given^a^	Recommendation Accepted, No. (%)
De-escalation	50	22 (44.0)
Discontinue	315	204 (65.8)
Redundant cover	37	9 (24.3)
Continue the same	763	704 (93.9)
Modify according to susceptibility	116	54 (46.6)
Escalation	7	6 (85.7)

a Numbers will not add up to the total number of patients because single patients may have received multiple recommendations.

**Table 6.** Reasons for Escalation and De-escalation of Antibiotic Therapy

Variable	No. (%)
**Reasons for escalation of antibiotic therapy**	
Modified according to culture and susceptibility	69 (56.2)
Worsening clinical condition	50 (43.8)
**Reasons for de-escalation of antibiotic therapy**	
Clinical improvement	166 (90.7)
Modified according to culture and susceptibility	17 (9.3)

**Table 7.** ASP Intervention Package

Antimicrobial Stewardship Intervention Package			
	2018	2019	2020
**Training**			
Training of physician selected in each hospital by providing a 1-year extensive fellowship in general infectious diseases	August 2018 to July 2019	August 2019	
Training of laboratory personnel as required in appropriate sample collection at testing at CMC Vellore	August 2018	May 2019	
Training of data entry operator on how to collect data, enter in Redcap and evaluate antimicrobial consumption using ASP metrics	August 2018		January 2020
**Intervention**			
Administrative support obtained from each of the 4 hospitals	February 2018		
ASP champion selected	March 2018		
Implementation of antibiogram **For antibiogram** Recording the results for all cultures and sensitivity for urine, sputum, fluid (please specify), pus and blood. Retrospectively collected data from past 1–3 years reports (minimum 30 isolates per specimen)		July 2019	
Implementation of antibiotic policy Data collected to prepare the policy **For antibiotic guidelines** • Antibiotics available • frequently used antibiotics • Discharge diagnosis for all patients (from discharge summary) • Surgery prophylaxis for each surgery • What type of surgery • What are the usual Infections encountered for patients after surgery		August 2019	
Setting up criteria for HAI surveillance based on the CDC guidelines and definitions for CLABSI, CAUTI, VAP, SSI		August 2019	
Implementation of ASP teams: trained physician fellow + IT support/data entry operator		August 2019	
Implementation of ASP intervention: prospective audit and feedback		August 2019	
**Evaluation**			
Antimicrobial consumption evaluated using ASP metric: days of therapy per 1,000 patient days	March 2018 August 2018		February 2020 to October 2021
Adherence to ASP recommendation provided by the team			
Evaluation of patient clinical outcomes			
Training of physician champion evaluated by series of assignments at the end of the course-evaluation exam		August 2019	

Note. ASP, antimicrobial stewardship program; HAI, healthcare-acquired infection; CDC, Centers for Disease Control and Prevention; CLABSI, central-line–associated bloodstream infection; CAUTI, catheter-associated urinary tract infection; VAP, ventilator-associated pneumonia; SSI, skin and soft-tissue infection.

### Secondary outcomes

The mortality rate was 3.7% during the baseline phase and 2.59% during the postintervention phase; the difference was not statistically significant (*P* = .193). Among the total deaths, infection-related mortality was 61% during the baseline phase and 43.8% in the postintervention phase.

The median length of hospital stay during the baseline phase was 5 days (IQR, 3–8) versus 6 days (IQR, 4–9) for the postintervention phase (*P* = .001). Adverse events were not observed in the baseline phase, but 7 episodes of diarrhea (0.6%) were reported in the postintervention phase (Table 4).

## Discussion

Several models exist for antimicrobial stewardship in LMICs, each with their own merits and demerits. A systematic review of interventions for ASP in low- and middle-income countries in 2021 elaborated upon single and multicomponent interventions.^11^ The most commonly utilized single-component interventions were education, training, guideline formulation, implementation, prescription auditing, and prospective audit with feedback. The predominant multicomponent interventions were combination of education and training followed by audit and feedback. Although education and training were easy to implement, the impact was not sustainable without constant reinforcement or supervision. Auditing of prescriptions showed the general trend of antibiotic use but provided no direct feedback in real time that could modify the behavior of the individual prescribers. In our study, we chose to implement a multipronged intervention approach that included a combination of education, training, guideline formulation, and laboratory augmentation, culminating in a prospective audit with feedback system.

Implementation of and sustaining a successful ASP in a low-resource setting has many barriers and challenges. A general lack of awareness regarding the short- and long-term implications of antimicrobial resistance among various senior administrators and healthcare workers has led to poor acceptance and implementation of an ASP. A lack of emphasis on rational antimicrobial prescribing in the undergraduate and postgraduate curricula is also a major lacuna, though recent efforts have been made to modify this. In the United States, a survey of medical schools revealed that antimicrobial stewardship was taught in only 66% of academic centers.^12^ Surprisingly, even among ID fellows, only half felt comfortable in leading stewardship programs at the completion of their training programs.^13^ A written assessment among practicing physicians found a singular lack of knowledge regarding appropriate antibiotic prescribing.^14^ Education and reinforcing optimal antibiotic use by senior and junior practicing physicians are key to improving antibiotic stewardship. Surveys reveal that >70% of prescribers were keen to improve their knowledge of antibiotics,^15^ suggesting that periodic training is useful. Very few structured training programs specifically cater to ASPs in India,^16^ although an attempt has been made by the Indian Medical Council to add ASPs to existing curricula.^16^ Training courses using interactive programs with case studies and prescribing methods are effective tools in ASP implementation, and often they are not part of even postdoctoral infectious diseases training programs.^13,14^ Thus, our customized ASP curriculum, with both online interactive modules and hands-on, case-based training of study physicians, demonstrated a significant reduction in antimicrobial usage in the postintervention phase. Training local physicians and pharmacists overcame the barrier of the absence of an infectious disease specialist and empowered them as ASP champions to lead their team in implementing a successful ASP.

Inadequate laboratory infrastructure and expertise also impair the ability of physicians to avoid empirical use, and to de-escalate or modify antibiotics according to susceptibility patterns. In addition, the nonavailability of laboratory information management systems in health settings can hamper the creation and development of an antibiogram. The Infectious Diseases Society of America emphasizes the critical role played by diagnostic stewardship and outlines 6 important components: a stratified antibiogram, cascade reporting, rapid viral testing for respiratory infections, rapid diagnostic serological tests, and serial monitoring of procalcitonin in ICU patients.^17,18^ However, these components may not be accessible or possible in all settings. A carefully phased adoption of these components could be cost-effective in the long run; hence, healthcare settings should be encouraged and mentored regarding their implementation. We were able to demonstrate this in our study: each center was assisted in developing their own antibiogram annually based on their own cultures using WHO-net with expertise from the main center.

In our study, common infectious syndromes noted in the secondary care in 75% of the cases were lower respiratory (25.2%), genitourinary (18.1%), and intra-abdominal infections (16.1%), followed by skin and soft-tissue infections (13.3%). Community-acquired infections predominated in both during the baseline phase and the postintervention phase (74.8% and 68.8%). The prevalences of MDR organisms in our study were 1.5% for carbapenem-resistant *Enterobacteriaceae* (CRE) in secondary-care hospitals and 57.4% in established tertiary-care centers, and for extended-spectrum β-lactamases (ESBLs) the prevalences were 9.6% in secondary-care hospitals versus 14.8% in established tertiary-care centers.^19^ Hence, in a secondary-care, low-resource setting, empiric use of high-end antibiotics can be restricted to microbiologically confirmed infectious disease syndromes in the community setting and in nosocomial infections. Both the WHO and the CDC recommend treatment guidelines as a priority for the judicious use of antibiotics, making it a critical component of any ASP program.^20,21^ Our project focused on creating treatment guidelines targeting specific infectious disease syndromes that are treated at these secondary-care hospitals considering their local antibiogram. Empirical antibiotics seemed to be justified in 80% of the cases at 48 hours in the postintervention arm, which was due to the implementation of a facility-based antibiotic policy and study physicians taking the lead in implementing the policy as well as performing a postprescription audit and feedback for specific areas in the healthcare setting.

Antibiotic consumption significantly decreased, with a marked reduction in use observed with cephalosporins, macrolides, and quinolones. However, an increase in β-lactam/β-lactamase inhibitor consumption was observed, possibly due to the higher rates of de-escalation and discontinuation of inappropriate use.^19^ No significant decrease in consumption of reserve antibiotics (carbapenem and colistin) was noted. Thus, cost and access prevented excess use when not indicated, proving that ASP efforts at secondary-care settings need to be directed toward the Access and Watch groups of antibiotics rather than the Reserve group.^22^ Overall, we achieved a significant improvement in optimal antibiotic use after our intervention.

Antibiotic-related adverse events and overall mortality were similar in both groups in our study, but infection-related mortality showed a trend toward favorable outcome in the postintervention group compared to the baseline group (43.8% vs 61%; *P* = .193), suggesting that optimizing antibiotic therapy does not lead to poor outcomes. A similar study in the United States also showed no difference in in-hospital mortality between preintervention and postintervention arms (11% and 14%; *P* = .44), suggesting that mortality did not increase with ASPs.^23^

Our study has proven that ASP can be successfully implemented with good outcomes in resource-limited, secondary-care hospitals by adopting a hub-and-spoke model. We leveraged freely available laboratory information management systems like WHO-net to create an antibiogram and to develop facility-specific antibiotic guidelines. We also augmented laboratory services through existing external quality control systems, and we trained ASP teams to recognize and treat infections through a blended training course that enabled them to do so with the least disruption to their routine busy clinical patient-care duties.

Thus, we created a sustainable intervention package that involves increasing local capacity building and creating skill sets both in the laboratory and clinical settings. This intervention led to a decrease in antibiotic consumption without any significant increase in mortality or morbidity and a decrease in infection-related mortality, improving the overall quality of care. This ASP model could be replicated with tertiary-care centers acting as nodal and support centers for multiple selected secondary-care centers for the implementation of ASPs.
